# Supplementary figures and images for: Spontaneous restoration of functional β‐cell mass in obese SM/J mice
Source: Physiol Rep. 2020 Oct 28;8(20):e14573. doi: 10.14814/phy2.14573 (PMC7592878; doi:10.14814/phy2.14573)

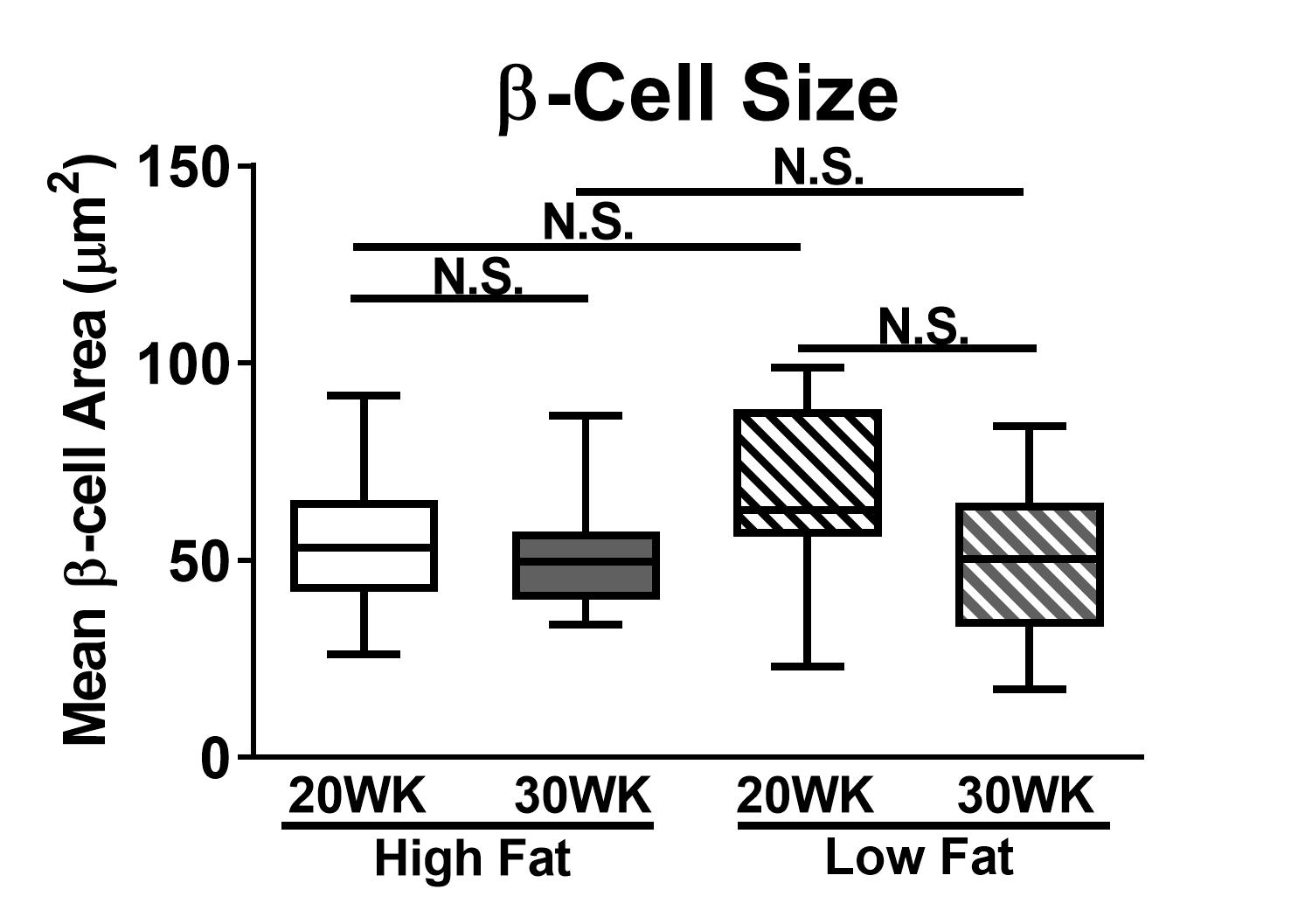

Supplement: Supplementary file 2 — Supplemental Figure 2 Final [file PHY2-8-e14573-s002.tif]

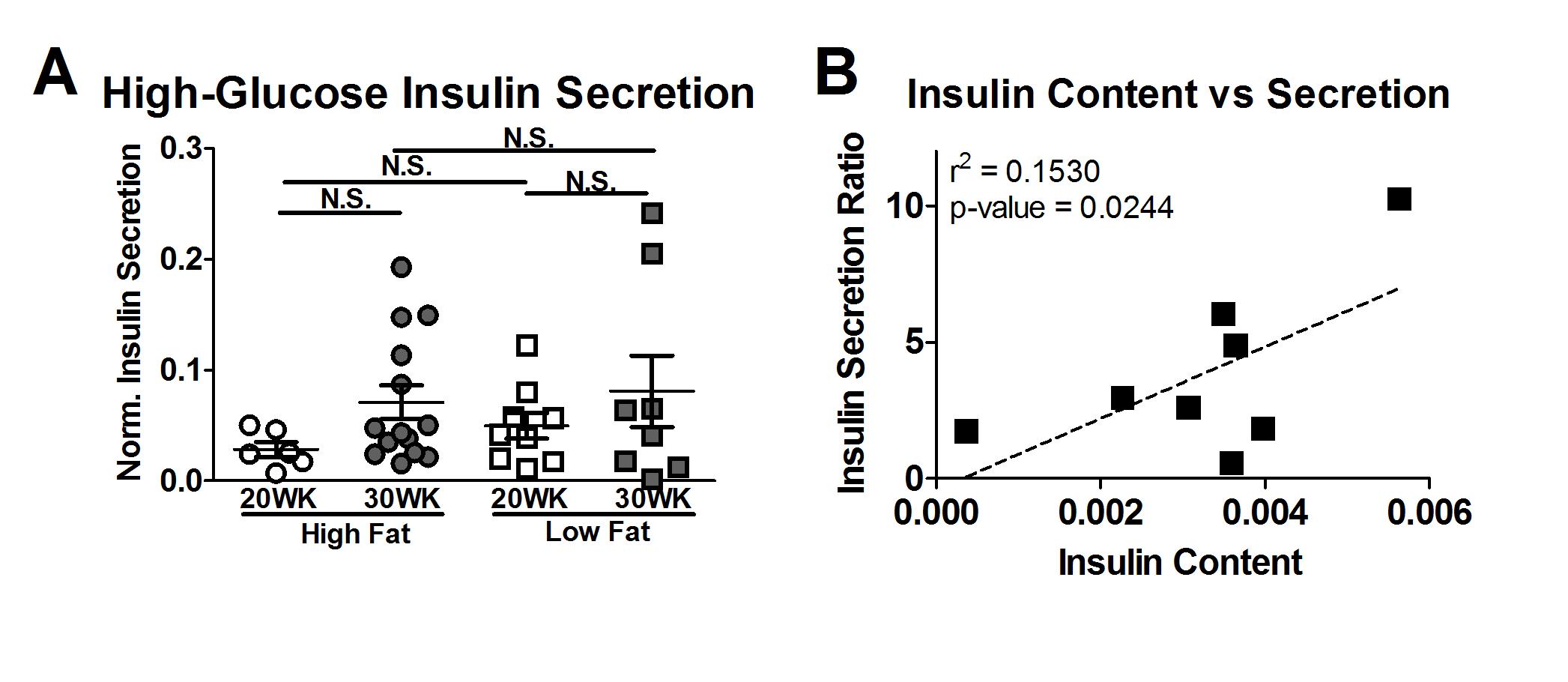

Supplement: Supplementary file 3 — Supplemental Figure 3 Final [file PHY2-8-e14573-s003.tif]
